# Artificial intelligence-assisted endoscopic diagnosis of esophageal squamous cell carcinoma

**DOI:** 10.3389/frai.2026.1837887

**Published:** 2026-09-09

**Authors:** Jie Mao, Kexun Li, Zilong Qian, Jianzhe Zhang, Shengguai Gao, GuoMin Tian, Daiheng Yang, Xin Tang, Xin Yang, Can Li, Yapeng Xing, Jian Xu, Chengwei Bi

**Affiliations:** 1 Department of Thoracic Surgery, Nanchong Central Hospital, Nanchong, Sichuan, China; 2 Key Laboratory of Lung Cancer of Yunnan Province, Department of Thoracic Surgery I, Yunnan Cancer Hospital, The Third Affiliated Hospital of Kunming Medical University (Yunnan Cancer Hospital), Kunming, China; 3 Department of Thoracic Surgery, Sichuan Clinical Research Center for Cancer, Sichuan Cancer Hospital and Institute, Sichuan Cancer Center, Affiliated Cancer Hospital of University of Electronic Science and Technology of China (Sichuan Cancer Hospital), Chengdu, China; 4 Urology Department Ward II, The Third Affiliated Hospital of Kunming Medical University, Kunming, Yunnan, China

**Keywords:** artificial intelligence, convolutional neural networks, early detection, endoscopic diagnosis, esophageal squamous cell carcinoma

## Abstract

Esophageal squamous cell carcinoma (ESCC) remains a major cause of cancer-related mortality, and prognosis depends strongly on detection at a curable stage. Endoscopy is central to screening and diagnosis, but subtle flat lesions, operator dependence, cognitive fatigue, lesion-location blind spots, and variability in interpretation contribute to missed or delayed diagnosis. Artificial intelligence (AI), particularly deep learning applied to white-light imaging, narrow-band imaging, blue-light imaging, magnifying endoscopy, Lugol chromoendoscopy, video endoscopy, and endocytoscopy, has shown clinically meaningful potential for lesion detection, margin delineation, invasion-depth estimation, and microvascular-pattern classification. Following peer-review feedback, this article has been reframed as a structured mini-review and evidence appraisal rather than a formal systematic review or meta-analysis. We summarize representative studies published mainly from 2019 to 2026, describe the literature-identification scope and eligibility criteria, and critically appraise the evidence using domains adapted from diagnostic-accuracy, prediction-model, and medical-imaging AI reporting frameworks. In this review, multimodal AI refers specifically to complementary endoscopic inputs rather than routine integration of histopathological or genomic data into deployed ESCC endoscopic systems. Current evidence suggests that many AI systems achieve high sensitivity in enriched image datasets, and several video-based, prospective, or randomized studies support translational feasibility. However, major limitations remain: most studies are retrospective; many use single-center or high-quality still-image datasets; confidence intervals, calibration, uncertainty quantification, subgroup analysis, failure-mode reporting, and latency benchmarks are inconsistent; and external validation across devices, operators, patient spectra, and live-video workflows remains limited.

## 1 Introduction

Esophageal cancer (EC) is a major global health burden. In East Asia, including China and Japan, ESCC is the predominant histological subtype and accounts for most EC cases (Siegel et al., 2023; Han et al., 2024). Although overall incidence rates vary by region and birth cohort, the absolute number of older patients remains substantial because of population aging (Lu et al., 2025). The survival gap between early and advanced disease is striking: early-stage ESCC can often be treated endoscopically with favorable outcomes, whereas advanced-stage disease carries a poor prognosis (Zhao et al., 2024; Pennathur et al., 2013). Therefore, improving early detection remains a high-value clinical objective.

Endoscopic screening and diagnosis rely on white-light imaging (WLI), chromoendoscopy, narrow-band imaging (NBI), blue-light imaging (BLI), magnifying endoscopy (ME), and endocytoscopy. Endoscopic screening has demonstrated population-level clinical benefit in randomized evidence (Liu et al., 2024). These modalities are complementary, but all are constrained by lesion subtlety, image quality, operator experience, withdrawal technique, and cognitive fatigue. Reported missed-lesion rates and post-endoscopy cancer events indicate that even high-quality endoscopy is not immune to error (Zhang, 2017; Chadwick et al., 2014; Zhou et al., 2025; Hassan et al., 2025; Zhang et al., 2020; Shimizu et al., 2008; Muto et al., 2010; Rodriguez de Santiago et al., 2019). AI-based image analysis has therefore been investigated as a second observer or decision-support tool to improve consistency in detection, characterization, and therapeutic planning. Prior AI diagnostic reviews and umbrella reviews have reported promising accuracy across endoscopic tasks, while also emphasizing heterogeneity, reporting limitations, and the need for robust validation (Zhang et al., 2021; Zha et al., 2024). This review focuses on AI-assisted endoscopic diagnosis of ESCC, including lesion detection, margin delineation, depth prediction, microvascular classification, multimodal integration, explainability, failure modes, and regulatory translation.

**Figure 1:**
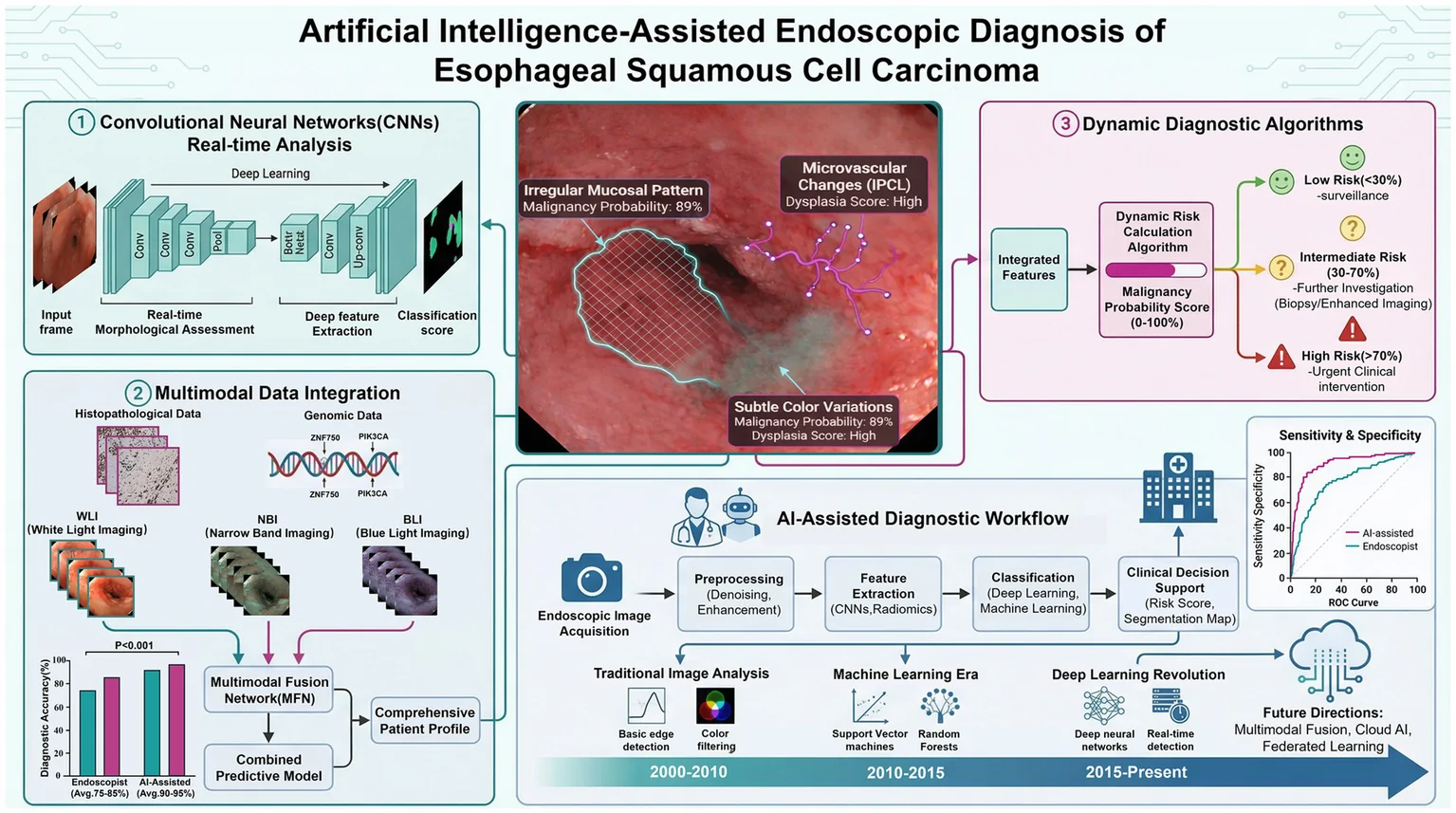
Virtual abstract of the structured mini-review and evidence appraisal of AI-assisted endoscopic diagnosis of esophageal squamous cell carcinoma.

This review is a structured mini-review and evidence appraisal designed for clinically oriented synthesis rather than quantitative evidence aggregation (Figure 1).

## 2 Review methodology and evidence-appraisal approach

### 2.1 Scope and literature-identification strategy

**Figure 2:**
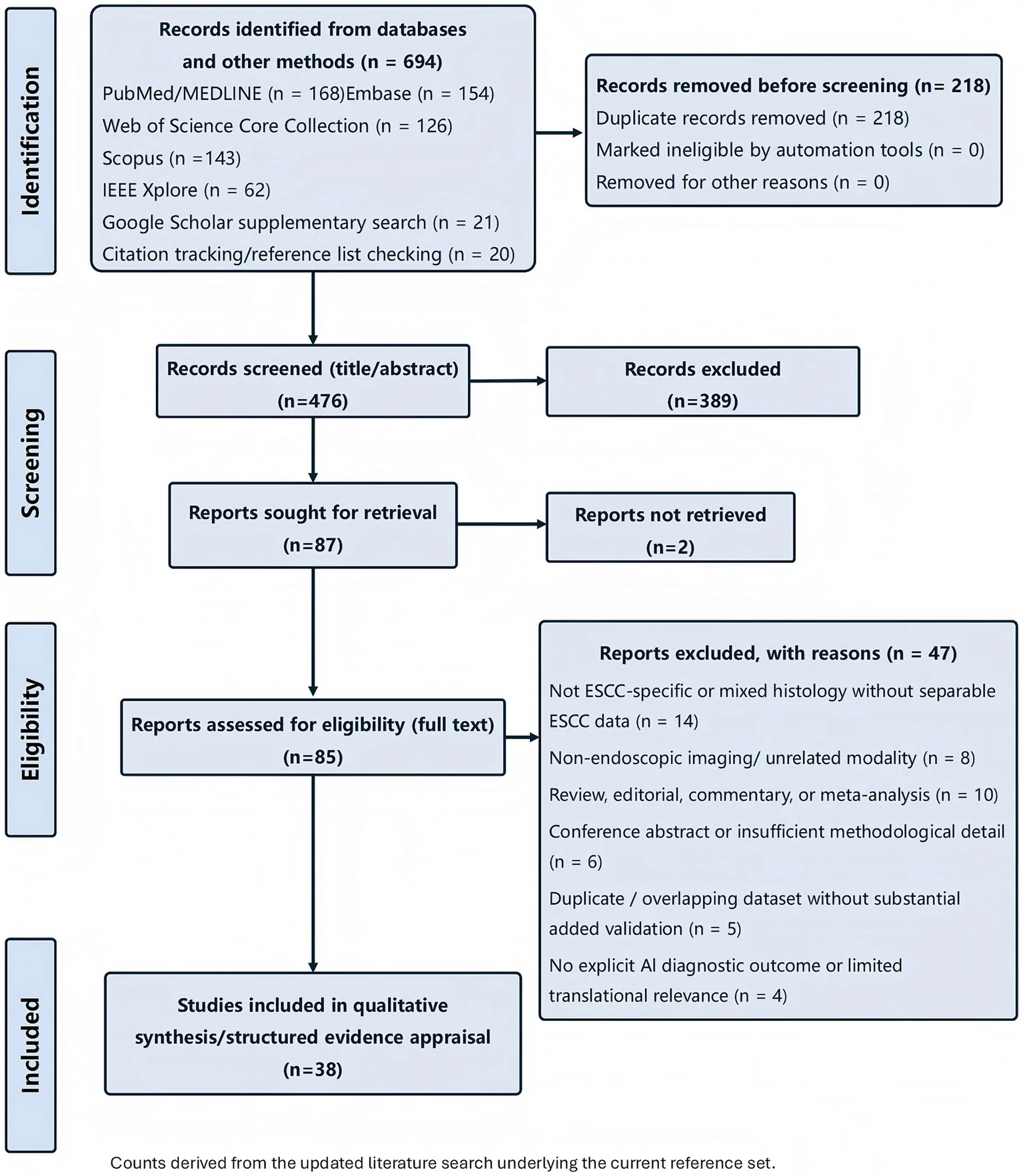
Flowchart of literature inclusion and screening.

The objective of this structured mini-review was to summarize and critically appraise AI-assisted endoscopic diagnosis of ESCC and squamous precancerous lesions. The review was informed by PRISMA 2020 transparency principles for search reporting (Page et al., 2021), while acknowledging that the article is not a registered systematic review and does not include pooled diagnostic-accuracy estimates. To reduce selection bias from a PubMed-only approach, the literature-identification strategy was broadened beyond PubMed/MEDLINE to include biomedical and engineering-oriented sources relevant to medical AI, including Embase, Web of Science Core Collection, Scopus, IEEE Xplore, Google Scholar, reference-list checking, and citation tracking of key ESCC AI studies. Search concepts combined disease, modality, and AI terms: “esophageal squamous cell carcinoma”, “oesophageal squamous cell carcinoma”, “precancerous lesion,” “high-grade intraepithelial neoplasia”, “endoscopy,” “white-light imaging,” “narrow-band imaging,” “blue-light imaging,” “Lugol,” “magnifying endoscopy,” “endocytoscopy,” “artificial intelligence,” “machine learning,” “deep learning,” “convolutional neural network”, “YOLO”, “single-shot multibox detector”, “transformer”, “vision transformer”, “computer-aided diagnosis”, “real-time”, “video”, “multimodal”, “explainable AI”, “self-supervised learning”, and “foundation model.” The primary focus was on studies published from January 2019 to May 2026, with older landmark studies retained when necessary for clinical or technical context (Figure 2).

### 2.2 Eligibility criteria

Studies were considered eligible if they evaluated AI, machine learning, or deep learning systems for endoscopic detection, classification, lesion localization, margin delineation, invasion-depth estimation, IPCL/microvascular classification, real-time video assistance, or multimodal endoscopic diagnosis of ESCC or squamous precancerous lesions. Eligible data sources included WLI, NBI, BLI, ME-NBI/BLI, Lugol chromoendoscopy, endocytoscopy, still images, video clips, and prospectively acquired endoscopic examinations. We included retrospective development/validation studies, external-validation studies, prospective observational studies, randomized trials, and clinically relevant technical studies with explicit performance metrics or translational relevance. Exclusion criteria were: non-endoscopic imaging only; esophageal adenocarcinoma without separable ESCC data; non-human or purely phantom studies; papers without identifiable AI methods or diagnostic outcomes; abstracts without sufficient methodological detail; duplicated reports from the same dataset unless a later study provided additional validation; and publications not relevant to clinical endoscopic diagnosis.

### 2.3 Data extraction, synthesis, and quality appraisal

For each representative study, we extracted the diagnostic task, imaging modality, model family, dataset composition, study design, validation design, unit of analysis, performance metrics, comparison with endoscopists, and translational limitations. Because modalities, case mix, annotation standards, outcome definitions, and thresholds differed markedly across studies, results were summarized narratively rather than pooled. When 95% confidence intervals (CIs) were unavailable in the source reports, the tables identify the limitation as “CI NR” or describe the absence of precision estimates rather than implying statistical certainty. Evidence quality and risk of bias were appraised narratively using domains adapted from QUADAS-2, CLAIM 2024, STARD-AI, CONSORT-AI, SPIRIT-AI, and TRIPOD+AI: patient/case selection, reference standard, index-test definition, separation of training and testing data, patient-level rather than image-level splitting, external validation, class balance, annotation process, robustness to artifacts and low-quality frames, calibration/uncertainty reporting, interpretability, real-time workflow evaluation, and post-deployment governance (Tejani et al., 2024; Sounderajah et al., 2025; Whiting et al., 2011; Liu et al., 2020; Rivera et al., 2020; Collins et al., 2024). To address the need for stronger methodological contextualization, we emphasized transparent source selection, extractable validation data, prespecified thresholds, external validation, model/code availability where feasible, and explicit reporting of bias, uncertainty, calibration, and clinical workflow context.

To make the evidence tables reproducible, studies were featured in Table 1 only when the source report provided, at minimum, an ESCC-relevant diagnostic task, endoscopic modality, model family, validation material or sample size, and at least one extractable performance benchmark such as sensitivity, specificity, accuracy, AUC, F1 score, PPV/NPV, or boundary-delineation accuracy. Table 2 was restricted to studies with video-based, real-time, prospective, randomized, or workflow-simulation evidence relevant to clinical translation. No absolute minimum sample size was imposed because several early ESCC AI studies are proof-of-concept investigations; instead, sample size, validation design, and missing precision estimates are displayed as methodological limitations. Studies without extractable validation material or diagnostic outcomes were excluded from the summary tables and discussed only narratively when they provided relevant technical context.

**Table 1:** Standardized summary of representative retrospective and validation studies in AI-assisted endoscopic diagnosis of ESCC.

Study	Task/modality	Architecture	Dataset/validation	Performance reported	Main limitation or risk-of-bias note
Horie et al. (2019)	WLI; EC/superficial vs. advanced cancer	CNN	8,428 images from 384 patients; retrospective	Accuracy 98%; all lesions <10 mm detected	Curated still images; limited benign controls; CI NR
Cai et al. (2019)	WLI; early ESCC screening	CNN-based CAD	2,428 training images; 187 validation images	Sensitivity 97.8%; specificity 85.4%; accuracy 91.4%; AUC > 0.96	Small validation set; controls mainly normal mucosa; CI NR
Tang et al. (2021)	WLI; early ESCC vs. reflux/normal	DCNN	4,002 training/cross-validation images; 1,033 validation images	Sensitivity 97.9%; specificity 88.6%; AUC 0.954	Multicenter validation but retrospective; device/operator shift not fully explored; CI NR
Feng et al. (2023)	WLI; superficial ESCC	CNN	5,892 training images; 4,529 validation images	AI assistance improved accuracy 75.12 to 84.95%	Retrospective; patient-spectrum and site-generalization concerns
Li et al. (2021)	NM-NBI versus WLI; early ESCC	CAD-NBI and CAD-WLI	2,167 abnormal and 2,568 normal NM-NBI images for training	CAD-NBI: sensitivity 91.0%; specificity 96.7%; accuracy 94.3%; CAD-WLI: sensitivity 98.5%; specificity 83.1%	Useful modality comparison; retrospective; CI NR
Wang et al. (2021)	WLI/NBI; histological grade classification	SSD	936 images; 264 test images	Tumor detection sensitivity 96.2%; specificity 70.4%; accuracy 90.9%; grading accuracy 92%	Pilot size; moderate specificity; benign mimics remain challenging
Everson et al. (2019)	ME-NBI; IPCL classification	CNN	7,046 images from 17 patients	Accuracy 93.7%; sensitivity 89.3%; specificity 98.0%	Proof-of-concept; very small patient count; CI ranges reported in limited form
Everson et al. (2021)	ME-NBI; clinically interpretable IPCL prediction	Interpretable CNN	67,742 high-quality ME-NBI images; expert comparison	CNN F1 94.0%; experts 97.0–98.0%	High image volume but still-image emphasis; dependence on expert-defined reference
Kumagai et al. (2019)	Endocytoscopy; ESCC diagnosis	GoogLeNet CNN	4,715 training images; 1,520 validation images	AUC 0.85 overall; sensitivity 92.6%; specificity 89.3%; accuracy 90.9%	ECS-specific; image quality and device availability limit generalization
Kumagai et al. (2022)	Endocytoscopy; modified type classification	DeiT transformer	7,983 training images; 114 test images from 38 cases	AUC 0.92; patient-level accuracy 94.7%	Small test set; limited direct comparison with CNNs
Ohmori et al. (2020)	WLI, NBI/BLI, ME; superficial ESCC detection	Deep neural network	9,591 non-ME and 7,844 ME training images	WLI: sensitivity 90.0%; specificity 76.0%; non-ME NBI/BLI: sensitivity 100.0%; specificity 63.0%; ME-NBI/BLI: sensitivity 98.0%; specificity 56.0%	High sensitivity but modest specificity; thresholds and modality-specific errors need analysis
Meng et al. (2022)	WLI/NBI; superficial ESCC/HGIN vs. benign	YOLOv5 CAD	Training: 622 ESCC/HGIN cases and 215 non-cancer cases; independent testing	AUC 0.982; accuracy 92.9%; sensitivity 91.9%; specificity 94.7%	Stronger benign-control inclusion; retrospective and dataset-specific
Liu et al. (2022)	WLI; lesion-margin delineation	Segmentation/localization AI	13,083 images; internal, external, and endoscopist comparison sets	Internal/external boundary accuracy 93.4%/95.7%	Boundary reference standard and interobserver variability should be explicit
Yuan X. L. et al. (2023)	NBI; detection and extent delineation	AI detection/delineation system	10,047 still images and 140 videos from four hospitals; prospective evaluation	Detection accuracy 92.4%/89.9% internal/external; delineation 88.9%/87.0%; prospective 91.4%/85.9%	Video and prospective data strengthen evidence; workflow and false-alert burden need further reporting
Nakagawa et al. (2019)	Non-ME/ME; invasion-depth classification	SSD	Training: 804 patients; validation: 155 patients	Sensitivity 90.1%; specificity 95.8%; PPV 99.2%; NPV 63.9%; accuracy 91.0%	Low NPV limits rule-out use; retrospective design
Shimamoto et al. (2020)	Video; invasion-depth assessment	Video AI system	23,977 images; 102 video clips; expert comparison	Sensitivity 71.0% vs. experts 50.0%; specificity 99.0% vs. experts 95.0% in one comparison	Video-based evidence; clip selection and latency/generalization require more detail
Urabe et al. (2025)	Histological surface images; invasive vs. non-invasive regions	Deep learning patch model	Surface histology images from early ESCC specimens	AUC 0.869; accuracy 78.8%	Mechanistic insight rather than direct endoscopic deployment

**Table 2:** Real-time, video-based, prospective clinical, and translational framework evidence relevant to ESCC AI translation.

Study	Clinical/technical setting	Validation material	Key finding	Translational limitation
Guo et al. (2020)	Real-time detection of precancerous lesions and early ESCC; NBI images and video clips	6,473 training images; 6,671 testing images; 80 video clips	Probability heatmaps closely matched endoscopist-marked areas	Requires prospective workflow validation and systematic false-positive analysis
Fukuda et al. (2020)	Real-time video detection and characterization of superficial ESCC; non-ME and ME-NBI/BLI	28,333 images from 862 videos; 144 short test clips	AI exceeded experts in detection sensitivity (91.0% vs. 79.0%) and characterization sensitivity (86.7% vs. 74.4%)	Specificity varied by modality; deployment latency and alert stability require reporting
Shimamoto et al. (2020)	Real-time video assessment of invasion depth	23,977 images; 102 video clips; comparison with 14 experts	Higher sensitivity and specificity than experts in selected comparisons	Clinical decision thresholds and external prospective impact remain unclear
Tajiri et al. (2022)	Simulated clinical classification of ESCC and non-cancerous esophageal lesions; NBI videos	25,048 ESCC images; 4,746 non-cancer images; 147 NBI clips	Accuracy 80.9%; sensitivity 85.5%; specificity 75.0%; exceeded endoscopist averages	Moderate specificity indicates false positives in benign conditions
Yang et al. (2021)	Real-time AI for early ESCC diagnosis	Clinical real-time endoscopy setting	Demonstrated feasibility of real-time assistance	Single-study workflow; standard latency/artifact benchmarks needed
Shiroma et al. (2021)	T1 ESCC detection from videos and effect of real-time assistance	Endoscopic videos	Reported video-detection feasibility and assistance effect	Video selection and patient-level clinical outcomes need standardization
Waki et al. (2021)	Detection of overlooked ESCC in videos simulating miss scenarios	Video-simulation evaluation	Supported use of AI as a second observer	Simulated setting may not represent true screening prevalence
Tani et al. (2023)	Prospective real-time usefulness study	Single-center prospective setting	Prospective workflow assessment	Single-center design limits generalizability
Yuan et al. (2024)	Multicenter tandem double-blind randomized trial of AI-assisted superficial ESCC/precancerous-lesion diagnosis	Multicenter real clinical setting; WLI and NM-NBI	Primary outcome: lesion and patient miss rate; trial completed	Among strongest translational evidence, but cost–benefit, implementation burden, frame-rate/latency reporting, and long-term outcomes remain to be assessed
Kelly et al. (2019)	Broader clinical-AI translation framework; not ESCC-specific	Narrative synthesis of clinical AI deployment challenges	Emphasizes prospective evaluation, representative test sets, clinically meaningful metrics, dataset shift, generalization, bias assessment, and post-market surveillance	Framework reference only; does not provide ESCC-specific diagnostic performance
Nakao et al. (2025)	Randomized controlled trial of an AI diagnostic system in clinical practice	Clinical-practice randomized design	Represents emerging RCT-level ESCC AI evidence	Details of performance by lesion subtype, device, and operator level should guide deployment

### 2.4 Methodological limitations of this review

This review has several methodological limitations that should be made explicit. First, because it is a structured mini-review rather than a registered systematic review, it does not provide duplicate independent screening statistics or meta-analytic pooling. Second, heterogeneity in primary-study design and reporting limits direct comparison: many studies report image-level metrics from enriched datasets, whereas clinical adoption requires patient-level and lesion-level outcomes in consecutive real-world procedures. Third, full reproducibility is limited because training code, trained weights, annotation manuals, negative-case composition, endoscope-device metadata, threshold-setting rules, and CIs are often unavailable; this caveat is consistent with AI reporting guidance that emphasizes complete model, data, validation, calibration, and uncertainty documentation (Tejani et al., 2024; Sounderajah et al., 2025; Collins et al., 2024).

## 3 Conventional endoscopic diagnosis and unresolved clinical needs

The standard diagnostic sequence for suspected early ESCC commonly begins with WLI or non-magnifying NBI/BLI to identify suspicious mucosal changes, followed by chromoendoscopy or ME-NBI/BLI to characterize lesion extent, microvascular morphology, and invasion risk. WLI is widely available and efficient but can miss subtle flat lesions or lesions with minimal color contrast. Lugol iodine staining increases sensitivity but has practical limitations, including longer procedure time, mucosal irritation, patient discomfort, and lower specificity in inflammatory or regenerative mucosa. ME-NBI improves visualization of IPCL patterns, yet interpretation depends heavily on training and experience (Hassan et al., 2025; Zhang et al., 2020; Shimizu et al., 2008; Muto et al., 2010; Rodriguez de Santiago et al., 2019; Inoue et al., 2015).

These clinical limitations create several AI-relevant use cases. First, a detection model could act as a real-time second observer for subtle lesions. Second, a classification model could reduce variability in distinguishing non-neoplastic inflammation from dysplasia or superficial cancer. Third, segmentation and heatmap models could support margin delineation before endoscopic resection. Fourth, invasion-depth prediction could inform the choice between endoscopic resection, surgery, and chemoradiotherapy. Finally, quality-control algorithms could identify blind spots, poor focus, excessive motion, or inadequate inspection time.

## 4 AI-assisted endoscopic diagnosis of ESCC

### 4.1 Single-modality image diagnosis

Most early ESCC AI studies used CNN-based architectures on still endoscopic images. WLI-based models demonstrated high sensitivity in retrospective datasets. Horie et al. trained a CNN on 8,428 EC images from 384 patients and reported 98% accuracy in distinguishing superficial from advanced cancer, with detection of small lesions under 10 mm (Horie et al., 2019). Cai et al. (2019) developed a WLI CAD system that achieved sensitivity of 97.8%, specificity of 85.4%, accuracy of 91.4%, and AUC greater than 0.96 in a validation set, improving performance especially among junior endoscopists. Tang et al. (2021) subsequently included reflux esophagitis and normal mucosa in multicenter validation and reported sensitivity of 0.979, specificity of 0.886, and AUC of 0.954. Feng et al. (2023) further reported a generalized WLI-based CNN system for superficial ESCC detection, showing that AI assistance improved diagnostic accuracy and specificity in endoscopist comparison experiments. These studies support diagnostic feasibility, but many were retrospective, used curated high-quality frames, and did not consistently report CIs, calibration, or patient-level generalization (Table 1).

NBI and BLI provide enhanced mucosal and vascular contrast. Li et al. (2021) compared CAD systems for NM-NBI and WLI and found that CAD-NBI achieved higher specificity and accuracy, whereas CAD-WLI showed higher sensitivity and negative predictive value. Wang et al. (2021) used SSD to classify histological grades from WLI and NBI images, reporting high sensitivity but only moderate specificity, highlighting the challenge of differentiating cancer from benign or inflammatory mimics. ME-NBI studies further focused on IPCL pattern recognition. Everson et al. demonstrated proof-of-concept CNN classification of normal and abnormal IPCL patterns and later expanded to a larger dataset, with performance approaching expert endoscopists (Everson et al., 2019; Everson et al., 2021). Other ME-NBI or microvascular studies have evaluated CAD, microvessel classification, and AI-assisted IPCL identification for early-stage ESCC (Zhao et al., 2019; Uema et al., 2021; Yuan et al., 2022b; Wang et al., 2023). Endocytoscopic studies using GoogLeNet and later DeiT further showed the potential of AI-assisted optical histology, but external validation and clinical workflow studies remain limited (Kumagai et al., 2019; Kumagai et al., 2022).

### 4.2 Multimodal endoscopic AI and fusion strategies

Multimodal AI is particularly relevant to ESCC because WLI, NBI, BLI, ME, iodine staining, endocytoscopy, still images, and continuous video emphasize different but complementary lesion features. In this review, multimodal should be understood as multimodal endoscopic imaging rather than combined endoscopy-histopathology-genomics modeling. Histopathology remains the reference standard for diagnosis and depth assessment, and genomic or molecular data may become valuable for future risk stratification, but the currently reviewed ESCC diagnostic systems are designed primarily for endoscopic image or video decision support. Ohmori et al. (2020) integrated WLI, NBI, BLI, and ME data and achieved high sensitivity across modalities, although specificity varied by modality. Meng et al. (2022) developed a YOLOv5-based CAD system for WLI and NBI that achieved AUC 0.982, accuracy 92.9%, sensitivity 91.9%, and specificity 94.7%, with improved performance among non-expert endoscopists when AI assistance was provided. Additional studies have expanded this direction through multiple endoscopic imaging modalities, YOLO-based clinical validation, hybrid modeling, transfer learning, and risk stratification for multiple Lugol-voiding lesions (Ikenoyama et al., 2021; Yuan et al., 2022a; Wang et al., 2022; Chou et al., 2023).

Technically, multimodal fusion can be organized at several levels. Early fusion concatenates aligned image channels or modality-specific feature maps before deep feature extraction, but it requires reliable registration and is vulnerable to missing or asynchronous modalities. Intermediate fusion uses parallel modality-specific encoders followed by shared attention, cross-attention, gated feature exchange, feature-pyramid integration, or modality-dropout training; this approach can preserve modality-specific information while learning complementary representations and robustness to absent modalities. Late fusion combines independent model outputs by voting, stacking, calibration-weighted averaging, or uncertainty-aware decision rules; it is simpler to deploy but may not fully exploit cross-modal interactions. For ESCC, temporal fusion across consecutive video frames is also important because a suspicious area may only be visible briefly during peristalsis, insufflation, or angle changes. General multimodal machine-learning frameworks distinguish early, intermediate, and late fusion and emphasize that fusion strategies should be chosen according to modality alignment, missing-modality robustness, and output-level calibration; ESCC models should therefore justify the level of fusion and report whether fusion improves robustness rather than only point accuracy (Baltrusaitis et al., 2019).

### 4.3 Lesion localization, margin delineation, invasion-depth estimation, and microvascular classification

Beyond binary detection, AI systems are increasingly designed to localize lesions and support therapeutic decision-making. Guo et al. (2020) generated probability heatmaps to delineate suspected lesion boundaries on NBI images and video clips. Yuan X. et al. (2023). reported real-time multimodal delineation of small flat-type ESCC. Liu et al. (2022) reported boundary-delineation accuracies above 90% in internal and external validation. Yuan X. L. et al. (2023) subsequently evaluated detection and delineation under NBI using still images and videos from multiple hospitals, with promising internal, external, and prospective performance. These studies are clinically important because incomplete lesion-margin assessment can affect endoscopic resection planning. However, boundary “truth” is not always straightforward; histopathology, expert consensus, iodine staining, and endoscopic resection specimens may disagree, and inter-annotator variability should be reported (Table 2).

Invasion-depth estimation is another high-impact task because submucosal invasion changes treatment strategy. Conventional IPCL-based depth assessment is clinically useful but has observer-dependence and reproducibility constraints (Sato et al., 2015). Nakagawa et al. (2019) used SSD-based modeling and reported sensitivity of 90.1%, specificity of 95.8%, and accuracy of 91.0% for distinguishing mucosal microinvasive cancer from submucosal invasive cancer. Related CNN-based and human-like ME-NBI systems have also been developed for ESCC invasion-depth prediction (Tokai et al., 2020; Zhang et al., 2023). Shimamoto et al. (2020) evaluated video-based invasion-depth assessment and reported higher sensitivity and specificity than expert endoscopists in some comparisons. Urabe et al. (2025) used AI to detect histological differences between invasive and non-invasive regions and showed that vascular features were associated with invasive patches. These findings suggest that AI may identify subtle morphologic and vascular correlates of depth, but negative predictive value, calibration, lesion selection, and decision thresholds require further validation before AI can guide definitive treatment selection.

### 4.4 Real-time video diagnosis and clinical deployment constraints

Real-time AI is the key translational step from retrospective image performance to clinical utility. Still-image datasets often exclude the most difficult frames: motion blur, defocus, bubbles, mucus, bleeding, overexposure, underexposure, peristalsis, folds, rapid scope movement, variable insufflation, and partial lesion visualization. Video-based models must therefore operate robustly under temporal noise and must distinguish true lesions from transient artifacts. Fukuda et al. trained an SSD-based model using images extracted from hundreds of videos and evaluated short video clips, reporting higher sensitivity than expert endoscopists for detection and characterization (Fukuda et al., 2020). Tajiri et al. (2022) evaluated an AI system on NBI video clips simulating clinical use and found improved accuracy and sensitivity compared with endoscopists, but specificity remained moderate. Additional real-time and video studies have examined early ESCC diagnosis, T1 ESCC video detection, and overlooked-lesion simulation, reinforcing both the feasibility and the need for standardized latency, artifact, and false-alert reporting (Yang et al., 2021; Shiroma et al., 2021; Waki et al., 2021).

Deployment also requires engineering, human-factors, and workflow validation. The model must process frames fast enough for the AI overlay to remain synchronized with endoscopic motion; bounding boxes or heatmaps should be temporally stable rather than flickering; false positives should not create alarm fatigue; and the interface must allow endoscopists to accept, reject, or override suggestions without disrupting inspection technique. Latency should be reported as an end-to-end quantity that includes image capture, preprocessing, inference, post-processing, display rendering, and network or hardware delays. Hardware integration differs across processors, endoscope vendors, image-enhancement modes, image resolutions, hospital networks, and computing accelerators. Continuous monitoring is needed because model drift can occur when devices, software versions, patient populations, procedural techniques, or training programs change. Prospective and randomized ESCC studies, including clinical-practice and multicenter tandem designs, are therefore more informative than image-level retrospective testing alone (Yuan et al., 2024; Tani et al., 2023; Nakao et al., 2025); broader clinical-AI translation guidance further emphasizes representative test sets, clinically meaningful endpoints, generalization, bias assessment, and post-market surveillance (Kelly et al., 2019). Broader randomized trials of AI-assisted gastric neoplasm and colon polyp detection provide additional translational precedent for workflow-integrated evaluation beyond offline image accuracy (Wu et al., 2021; Wang et al., 2019).

### 4.5 CNNs, transformers, and hybrid architectures

CNNs remain the dominant architecture in ESCC endoscopic AI because they efficiently learn local texture, color, vascular, and edge features and can be adapted to detection frameworks such as SSD and YOLO. Their locality bias and parameter efficiency are advantageous when datasets are relatively small, as is common in medical imaging. However, CNNs can be limited in modeling long-range spatial dependencies, global context, and relationships between non-contiguous mucosal regions. Transformer-based models, including ViT, DeiT, and Swin Transformer variants, use self-attention to model global dependencies and may better capture context across a broad field of view, but they are data-hungry, computationally demanding, and more prone to overfitting when training cohorts are small or device-specific (Kumagai et al., 2022; Shamshad et al., 2023; Li et al., 2023). General multimodal and transformer literature provides useful methodological vocabulary, but domain-specific gains cannot be extrapolated to ESCC without direct endoscopic validation (Baltrusaitis et al., 2019; Shamshad et al., 2023; Li et al., 2023).

Only limited ESCC-specific evidence directly compares CNNs and transformers under identical data partitions. Kumagai et al. reported a DeiT-based endocytoscopic system with AUC 0.92 and patient-level accuracy 94.7%, suggesting feasibility but not proving architectural superiority (Kumagai et al., 2022). In broader medical imaging, transformer and hybrid CNN-transformer architectures are increasingly used for classification, segmentation, and multimodal diagnosis (Shamshad et al., 2023; Li et al., 2023). Reporting guidance for medical-imaging AI and AI prediction models shows why architectural comparisons should include external validation, locked testing, calibration and uncertainty, reproducibility, and deployment information, not only accuracy (Tejani et al., 2024; Collins et al., 2024). For ESCC, the most plausible near-term direction is hybrid design: CNN stems for efficient local texture extraction, attention modules for global context, temporal modules for video stability, and uncertainty-aware heads for calibrated clinical output; hybrid transfer-learning studies in esophageal endoscopic detection are therefore particularly relevant (Chou et al., 2023). Claims that transformers outperform CNNs should be reserved for studies using the same patient-level external test sets, the same threshold-locking rules, and the same real-time hardware constraints.

Architecture selection should therefore be matched to the diagnostic use case rather than listed as interchangeable model names. Standard CNN patch-classifiers are well suited to offline still-image classification, optical-histology classification, or confirmation of cropped regions because they can learn local color, texture, and vascular features with relatively modest data requirements. However, patch classifiers usually require a separate candidate-generation or sliding-window step and may process only selected frames; this can increase end-to-end latency and create unstable outputs when the endoscope is moving. By contrast, one-stage detectors such as YOLO and SSD predict class probabilities and bounding boxes in a single forward pass, which makes them more appropriate for live video streams where the system must process sequential frames, maintain a stable overlay, and avoid perceptible delay. Their speed advantage is clinically meaningful only when reported as end-to-end FPS and latency on the actual deployment hardware, including capture, preprocessing, inference, post-processing, display rendering, and any network transmission. The trade-off is that aggressive model compression, lower input resolution, or high detection thresholds may miss very small, flat, or partially visualized lesions, whereas segmentation networks may better support margin delineation but often require heavier computation and denser annotation.

The same speed-accuracy trade-off should be handled as a reporting requirement rather than as an architecture claim. For live ESCC workflows, lightweight backbones, feature-pyramid or multiscale modules, temporal smoothing, and one-stage detector heads may reduce latency, but their clinical value depends on locked validation in artifact-rich videos and transparent reporting of end-to-end performance. Future ESCC studies should therefore report parameter count, model size, FLOPs, input resolution, FPS, hardware accelerator, and latency alongside diagnostic accuracy, so that clinicians can judge whether a model is suitable for dynamic endoscopy rather than only for retrospective image benchmarking (Tejani et al., 2024; Collins et al., 2024; Kelly et al., 2019).

### 4.6 Dataset quality, annotation variability, and class imbalance

Dataset construction is a central determinant of AI validity. Many ESCC datasets overrepresent clear cancer images and underrepresent real-world negative or confounding conditions such as reflux esophagitis, radiation injury, post-endoscopic resection scars, varices, glycogenic acanthosis, candidiasis, vascular ectasia, submucosal lesions, white plaques, and benign Lugol-voiding areas. Models trained on such enriched data may show inflated sensitivity and specificity but fail under screening conditions where disease prevalence is low and benign abnormalities are common.

Annotation is also complex. Detection labels may be lesion-level, frame-level, or pixel-level; segmentation labels may be drawn from expert consensus, iodine staining, ME-NBI findings, or post-resection pathology; and invasion-depth labels depend on histopathological sampling and classification thresholds. Interobserver variability should be quantified using agreement statistics, and discordant labels should be adjudicated transparently. Patient-level splitting is essential to avoid leakage from near-duplicate frames or multiple frames from the same lesion appearing in both training and testing sets. Class imbalance should be addressed through transparent sampling, loss-function design, threshold tuning, and evaluation under clinically plausible disease prevalence. Future studies should report not only sensitivity, specificity, accuracy, and AUC, but also PPV, NPV, lesion miss rate, false alerts per procedure, calibration metrics, decision-curve analysis, and 95% CIs at patient or lesion level. Comparable practices are needed for ESCC endoscopic datasets, including transparent data provenance, locked test sets, external validation, and availability of code, models, or reproducible implementation details when feasible (Tejani et al., 2024; Collins et al., 2024).

### 4.7 Interpretability and explainable AI

Clinical trust requires more than high point estimates. Heatmaps, Grad-CAM, saliency maps, attention visualization, prototype-based explanations, uncertainty estimates, and counterfactual analysis can help clinicians understand which image regions influenced model output. For ESCC, interpretable outputs are most useful when they correspond to known endoscopic features such as demarcation lines, IPCL irregularity, brownish areas, microvascular changes, flat depressed morphology, or Lugol-voiding margins. However, explainability methods can be visually persuasive without being faithful to model reasoning. Broader critiques of explainable AI in health care caution that visually persuasive heatmaps or post-hoc explanations should not be treated as evidence of safety or faithful model reasoning (Ghassemi et al., 2021). XAI outputs should therefore be validated against expert lesion and margin annotations, tested for stability under image perturbation, and reported together with uncertainty or reject-option mechanisms. Interpretability should support—but never substitute for—prospective clinical validation.

### 4.8 Failure modes and safeguards

Failure-mode analysis is underreported in the ESCC AI literature. Potential false negatives include very small flat lesions, lesions at the upper esophageal sphincter or near folds, partially visualized lesions, low-contrast lesions under WLI, lesions obscured by mucus or bubbles, and frames affected by motion blur, poor focus, bleeding, overexposure, underexposure, or rapid scope withdrawal. Potential false positives include reflux esophagitis, radiation injury, inflammation, scars, glycogenic acanthosis, candidiasis, white plaques, vascular congestion, varices, benign submucosal lesions, and iodine-unstained non-neoplastic mucosa. False localization can occur when heatmaps highlight specular reflection, shadows, borders of folds, bubbles, or non-lesion texture rather than true pathologic tissue. Broader clinical-AI translation and robustness literature reinforces the need to describe rare but clinically consequential failure modes, including artifacts, domain shift, lesion mimics, unintended bias, and adversarial or spurious visual cues, rather than reporting average-case performance alone (Kelly et al., 2019; Finlayson et al., 2019).

Several mitigation strategies are being actively explored for these failure modes. Technical approaches include hard-negative mining with reflux, scar, radiation-injury, candidiasis, and specular-reflection examples; artifact-aware augmentation that simulates motion blur, defocus, mucus, bubbles, bleeding, variable illumination, and partial visualization; frame-quality classifiers that suppress or flag unusable frames; temporal smoothing, optical-flow tracking, or recurrent/transformer memory to require persistence across consecutive frames before an alarm is displayed; uncertainty-aware calibration and reject options for low-confidence predictions; and domain adaptation or federated learning to reduce device- and center-specific drift. For margin and depth tasks, combining detection with segmentation heatmaps, IPCL-aware features, and modality-specific confirmation under NBI/BLI or Lugol staining may reduce false localization and improve clinically interpretable outputs.

Clinical safeguards are equally important. Endoscopists can mitigate false negatives by cleaning mucus and bubbles, optimizing insufflation and focus, slowing withdrawal at the upper esophageal sphincter and near folds, switching between WLI and image-enhanced modes for suspicious low-contrast areas, and using AI as a second observer rather than as a replacement for systematic inspection. Recommended deployment safeguards include external validation across institutions and devices, stress testing with artifact-rich videos, stratified performance reporting by lesion size, morphology, location, and imaging modality, uncertainty thresholds or reject options, human override, audit logs, incident reporting, post-market monitoring, and periodic model updating under change-control governance. Such safeguards are consistent with broader clinical-AI translation, robustness, and regulatory lifecycle recommendations (Kelly et al., 2019; Finlayson et al., 2019; International Medical Device Regulators Forum, 2017; U.S. Food and Drug Administration, 2025; European Parliament and Council of the European Union, 2024; World Health Organization, 2021; U.S. Food and Drug Administration, 2021). Clinicians must retain responsibility for final diagnosis, biopsy/resection decisions, management of discordant AI-human judgments, and patient communication (Table 3).

**Table 3:** Narrative quality and risk-of-bias appraisal using domains adapted from QUADAS-2, CLAIM, STARD-AI, CONSORT-AI, SPIRIT-AI, and TRIPOD+AI.

Domain	Observed status in ESCC AI literature	Risk/applicability concern	Implication for this review and future studies
Search and selection transparency	Earlier ESCC reviews often relied heavily on PubMed and narrative selection.	Selection bias; incomplete coverage of engineering literature and conference-derived AI methods.	Search scope, databases, terms, inclusion/exclusion criteria, and non-meta-analytic design are now stated; reproducibility-related reporting expectations are explicitly acknowledged (Collins et al., 2024).
Patient/case selection	Many studies use enriched image datasets with high disease prevalence and high-quality frames.	Spectrum bias; performance may be overestimated relative to screening.	Interpret results as feasibility evidence; prioritize patient-level external validation.
Reference standard	Histology is often used, but margin and invasion labels may vary by biopsy, resection specimen, iodine staining, and expert consensus.	Mislabeling and verification bias; uncertain ground truth for boundaries.	Report reference-standard hierarchy and interobserver agreement.
Training/testing separation	Some papers report image-level splits; multiple frames from one lesion can be near-duplicates.	Data leakage if patient-level separation is not enforced.	Require patient-level and center-level separation for testing.
External validation	External and multicenter validation is increasing but remains inconsistent.	Domain shift across endoscope vendors, processors, centers, operators, and patient populations.	Use multicenter external test sets and prospective deployment monitoring.
Metrics and precision	Sensitivity, specificity, accuracy, and AUC are commonly reported, but CIs, calibration, and false alerts per procedure are inconsistently available.	Uncertain precision and clinical utility; threshold-dependent results.	Tables define CI NR when unavailable; future studies should report 95% CIs, calibration, decision thresholds, and false-alert burden.
Class imbalance and benign mimics	Negative controls may be normal mucosa only; inflammatory and scar-like mimics may be underrepresented.	False positives and false reassurance under real-world prevalence.	Include clinically plausible negative-case spectra and benign mimics; report class balance, sampling strategy, prevalence assumptions, PPV/NPV under expected screening prevalence, and subgroup performance rather than relying on enriched image-level accuracy alone.
Video and real-time robustness	Still-image performance may not survive motion, blur, mucus, bubbles, lighting changes, and rapid scope movement.	Reduced real-time reliability and alert fatigue.	Stress-test with real procedure videos and report end-to-end latency, frame rate, false alerts per procedure, overlay stability, artifact-specific performance, failure-mode taxonomies, and monitoring for domain shift or spurious visual cues (Kelly et al., 2019; Finlayson et al., 2019).
Interpretability	Heatmaps and attention maps are sometimes shown but rarely validated quantitatively.	Misleading explanations can increase unwarranted trust.	Validate XAI outputs against expert lesion/margin annotations and combine heatmaps, attention maps, post-hoc explanation audits, and uncertainty estimation; visually persuasive explanations should be audited rather than accepted at face value (Ghassemi et al., 2021).
Clinical impact	Prospective trials are emerging but remain fewer than retrospective studies.	Unclear effect on miss rate, procedure time, biopsy/resection decisions, cost, and training.	Use prospective, randomized, and workflow-integrated studies with predefined clinical outcomes, including lesion miss rate, procedure time, biopsy/resection decisions, false-alert burden, training effect, cost-effectiveness, and unintended consequences (Kelly et al., 2019; Vasey et al., 2022).
Regulatory and ethics	Data governance, privacy, cybersecurity, post-market monitoring, and liability are variably discussed.	Barriers to deployment and patient trust.	Treat diagnostic AI as regulated SaMD; define human oversight, audit trails, model-update governance, and safety monitoring.

### 4.9 Regulatory, ethical, and implementation considerations

AI endoscopic systems intended for diagnosis or clinical decision support will generally be regulated as software as a medical device or as part of an integrated medical device, depending on jurisdiction and intended use. Regulatory expectations include analytical validity, clinical validity, usability, cybersecurity, risk management, human oversight, post-deployment monitoring, change-control plans for adaptive models, and transparent labeling of intended use. International and regional frameworks emphasize clinical evaluation of SaAI/ML device oversight, predetermined change-control planning, responsible governance for AI in health, and risk-based obligations for high-impact clinical AI systems (International Medical Device Regulators Forum, 2017; U.S. Food and Drug Administration, 2025; European Parliament and Council of the European Union, 2024; World Health Organization, 2021; U.S. Food and Drug Administration, 2021).

Ethically, ESCC AI systems must avoid widening disparities. Training and validation datasets should represent different patient populations, endoscope platforms, operator skill levels, clinical settings, and screening-risk profiles. Privacy-preserving data sharing, federated learning, secure annotation platforms, and de-identification pipelines may help expand multicenter datasets without exposing patient data. Liability should be defined for scenarios in which AI misses a lesion, generates excessive false positives, or influences an inappropriate clinical decision. Implementation should also address consent, transparency to patients where required, cybersecurity, auditability, fairness monitoring across subgroups, and clinician deskilling risk. These considerations are essential for responsible translation from retrospective image performance to safe clinical use.

## 5 Future directions

The next phase of ESCC AI development should move from proof-of-concept image classification toward validated clinical systems. First, multicenter prospective datasets should capture consecutive procedures rather than selected images. Metadata should include endoscope model, processor, imaging mode, resolution, frame rate, operator experience, lesion size, location, morphology, histology, treatment, and follow-up. Second, benchmarking should standardize patient-level, lesion-level, and frame-level metrics; report 95% CIs; separate tuning, validation, and locked testing; and include calibration, false alerts per procedure, time-to-detection, and decision thresholds. Third, real-time trials should measure workflow effects, including procedure duration, endoscopist attention, biopsy decisions, lesion miss rate, patient outcomes, cost-effectiveness, and unintended consequences such as over-biopsy or alert fatigue. These priorities are aligned with recent calls for transparency, reproducibility, workflow-integrated evaluation, explainable outputs, and explicit failure-mode reporting in healthcare AI (Tejani et al., 2024; Collins et al., 2024; Kelly et al., 2019; Vasey et al., 2022; Ghassemi et al., 2021; Finlayson et al., 2019).

Methodologically, self-supervised learning and foundation-model approaches may reduce dependence on expensive pixel-level annotations by learning representations from large unlabeled endoscopic video archives (Azizi et al., 2021). Such models could be fine-tuned for ESCC detection, margin delineation, depth prediction, microvascular classification, and quality control. Hybrid CNN-transformer networks, temporal transformers, multimodal cross-attention, federated learning, domain adaptation, synthetic minority oversampling, and uncertainty-aware calibration are also promising. At the same time, clinical deployment requires explicit attention to model efficiency and lifecycle governance: lightweight backbones, feature-pyramid or multiscale modules, temporal smoothing, and detector heads may help balance parameter count, multiscale feature capture, and real-time FPS in ESCC video workflows, but they must be validated under locked testing, documented change-control procedures, and post-deployment monitoring (Kelly et al., 2019; U.S. Food and Drug Administration, 2021). Nevertheless, technical novelty should be subordinated to clinical validity: a simpler model with transparent external validation and stable real-time performance may be preferable to a complex model that lacks generalization (Table 4).

**Table 4:** Recommended reporting, benchmarking, and deployment checklist for future ESCC AI studies.

Domain	Minimum information to report	Why it matters for ESCC AI	Reviewer concern addressed
Study design	Retrospective/prospective design; single- or multicenter setting; consecutive versus enriched sampling; patient-, lesion-, and image-level units; prespecified threshold-locking rule; early clinical-evaluation stage and human-factor context where applicable (Vasey et al., 2022).	Clarifies whether reported performance reflects real screening workflow or curated image feasibility.	Methodological transparency and reproducibility (Collins et al., 2024)
Dataset composition	Endoscope vendor, processor, imaging mode, resolution, frame rate, lesion size/location/morphology, benign mimics, negative-case spectrum, class balance, and cross-dataset validation strategy; broader medical-image AI examples should be interpreted with disease-domain differences in mind (Tejani et al., 2024; Collins et al., 2024).	Prevents overestimation from high-quality cancer-enriched datasets and supports domain-shift analysis, while clarifying whether external datasets truly reflect ESCC screening conditions.	Dataset quality, class imbalance, and generalizability
Reference standard and annotation	Histology source; biopsy versus resection specimen; expert consensus method; number and experience of annotators; interobserver agreement; adjudication of discordant labels.	Margin, IPCL, and invasion-depth labels are clinically complex and can introduce verification or labeling bias.	Inclusion criteria, annotation variability, and risk of bias
Model and training details	Architecture family; use-case mapping (classification, detection, segmentation, depth prediction, or quality control); preprocessing; augmentation; hyperparameters; training/validation/test split; patient-level leakage prevention; calibration method; parameter count, model size, FLOPs, input resolution, FPS, hardware environment, code/model availability or reasons for restricted sharing, and change-control assumptions for deployed models (Tejani et al., 2024; Collins et al., 2024; U.S. Food and Drug Administration, 2021).	Allows meaningful comparison among CNN, transformer, YOLO/SSD, segmentation, lightweight, and hybrid systems and supports reproducibility; multimodal designs should report fusion strategy, missing-modality handling, and cross-modal contribution (Baltrusaitis et al., 2019).	Architecture comparison, model-size trade-offs, multiscale feature capture, real-time feasibility, and reproducibility
Performance metrics	Sensitivity, specificity, accuracy, AUC, PPV, NPV, lesion miss rate, false alerts per procedure, calibration, decision-curve analysis, and 95% CIs at patient or lesion level.	Point estimates alone are insufficient for clinical adoption, especially under low-prevalence screening conditions.	Statistical precision and standardized benchmarking
Real-time deployment	End-to-end latency, FPS, overlay stability, hardware accelerator, memory footprint, artifact stress testing, motion/blur/mucus/bubble performance, user-interface design, temporal smoothing rules, lesion-detection failure-mode taxonomy, and post-deployment monitoring plan (Kelly et al., 2019; Finlayson et al., 2019; U.S. Food and Drug Administration, 2021).	Retrospective still-image accuracy may not translate to live endoscopy; robustness failures can arise from artifacts, benign mimics, device shift, transient partial lesion visualization, spurious visual cues, or insufficient frame-rate handling (Kelly et al., 2019; Finlayson et al., 2019).	Real-time AI diagnosis, robustness, and deployment constraints
Interpretability and safeguards	Heatmap/XAI validation against expert annotations; post-hoc explanation audit where applicable; uncertainty estimates; reject options; human override; audit logs; and safeguards for discordant AI-human judgments (Ghassemi et al., 2021; U.S. Food and Drug Administration, 2021).	Clinicians need actionable explanations and safety mechanisms for uncertain or discordant predictions; XAI outputs should be treated as testable explanations rather than proof of model reasoning (Ghassemi et al., 2021).	Explainability and clinical safeguards
Regulatory and ethics	Intended use, SaMD status, privacy and cybersecurity controls, subgroup fairness, post-market monitoring, predetermined change-control plan, model-update governance, and liability pathway (International Medical Device Regulators Forum, 2017; U.S. Food and Drug Administration, 2025; European Parliament and Council of the European Union, 2024; World Health Organization, 2021; U.S. Food and Drug Administration, 2021).	Responsible deployment requires safety, accountability, and continuous monitoring beyond initial accuracy testing.	Regulatory and ethical considerations

Implementation should follow a human-centered pathway. AI output should be designed for endoscopist comprehension, with adjustable alert thresholds, stable visual cues, clear confidence information, and easy override. Training programs should teach clinicians both how to use AI and how to recognize its limitations. Hospitals should define responsibility for local validation, software updates, adverse-event review, and model-performance surveillance. Post-market monitoring should track performance drift, failure modes, false-alert burden, missed lesions, cybersecurity incidents, and equity across patient subgroups.

## 6 Conclusion

AI-assisted endoscopic diagnosis of ESCC has progressed rapidly from retrospective still-image classification to multimodal endoscopic, video-based, and prospective clinical evaluation. Current evidence indicates that AI can improve sensitivity in selected datasets and may support less-experienced endoscopists, especially for subtle superficial lesions and precancerous changes. However, current evidence is limited by retrospective design, dataset enrichment, single-center development, inconsistent CI reporting, insufficient calibration and failure-mode analysis, and incomplete evaluation of real-time deployment constraints such as model size, frame-rate stability, and end-to-end latency. AI should therefore be implemented as an assistive second observer under clinician oversight rather than as an autonomous diagnostic authority.

## Glossary

EC: esophageal cancer
ESCC: esophageal squamous cell carcinoma
AI: artificial intelligence
CNN: convolutional neural networks
WLI: white light imaging
ME: magnifying endoscopy
NBI: narrow-band imaging
YOLO: you only look once
SSD: single-shot multibox detector
BLI: blue light imaging
CAD: computer-aided detection
AUC: area under the receiver operating characteristic curve
DCNN: deep convolutional neural network
NM-NBI: non-magnified narrow-band imaging
ME-NBI: magnifying narrow-band imaging
IPCL: the intrapapillary capillary loop
ESC: the endocytoscopic system
DeiT: the vision transformer, data-efficient image transformer
HGIN: high-grade intraepithelial neoplasia
HrEL: high-risk esophageal lesion
FPN: feature pyramid network
FPS: frames per second
FLOPs: floating-point operations
SaMD: software as a medical device
